# Evaluation of sixty-eight cases of fracture stabilisation by external hybrid fixation and a proposal for hybrid construct classification

**DOI:** 10.1186/s12917-014-0189-4

**Published:** 2014-09-20

**Authors:** María Jiménez-Heras, Gian Luca Rovesti, Gianluca Nocco, Massimo Barilli, Paolo Bogoni, Emilio Salas-Herreros, Matteo Armato, Francesco Collivignarelli, Federico Vegni, Jesus Rodríguez-Quiros

**Affiliations:** Centro Veterinario Eurocan, Guadarrama, Madrid Spain; Clinica Veterinaria M. E. Miller, Cavriago, (RE) Italy; Centro Specialistico Veterinario di Lecce, Lecce, Italy; Ospedale Veterinario “I Portoni Rossi”, Zola Pedrosa, (BO) Italy; Ambulatorio Veterinario Associato Bogoni & Pasotti, Ghedi, (BS) Italy; Hospital Veterinario Benartemi 24H, Vecindario, Gran Canaria Spain; Fundacion Hospital ClinicoVeterinario de Castilla y Leon, Leon, Spain; Clinica Veterinaria Roma Sud, Roma, Italy; Ambulatorio Veterinario Casentino, Poppi, (AR) Italy; Department of Internal Medicine and Surgery, Veterinary Teaching Hospital, Veterinary School, Complutense University, Madrid, Spain

**Keywords:** Fracture, External hybrid fixation, Hybrid frame classification, Dog, Cat

## Abstract

**Background:**

Hybrid external fixation (HEF) is an emerging technique for fracture stabilization in veterinary orthopedics, but its use has been reported in few papers in the veterinary literature. The linear and circular elements that form hybrid fixators can be connected in a very high number of combinations, and for this reason just referring to HEF without any classification is often misleading about the actual frame structure. The aim of this study was to retrospectively evaluate fracture stabilization by HEF in 58 client-owned dogs and 8 cats, and to extend the already existing classification for hybrid constructs to include all frame configurations used in this study and potentially applicable in clinical settings. Animal signalment, fracture classification, surgical procedure and frame configuration were recorded. Complications, radiographic, functional and cosmetic results were evaluated at the time of fixator removal.

**Results:**

Sixty-eight fractures in 58 dogs and eight cats were evaluated. Two dogs had bilateral fractures. Fifty-one percent were radio-ulna, 34% tibial, 9% humeral, 3% femoral and 3% scapular fractures. One ring combined with one or two linear elements was the most widely employed configuration in this case series. Radiographic results at the time of frame removal were excellent in 59% of the cases, good in 38% and fair in 3%, while functional and cosmetic results were excellent in 69% of the cases, good in 27% and fair in 4%.

**Conclusions:**

HEF is a useful option for fracture treatment in dogs and cats, particularly for peri and juxta-articular fractures. It can be applied with a minimally invasive approach, allows adjustments during the postoperative period and is a versatile system because of the large variety of combinations that can fit with the specific fracture features. The classification used enables to determine the number of linear and circular elements used in the frame.

## Background

External fixation is a very commonly used technique for fracture stabilisation. It can be used in open or closed fractures, produces minimal tissue disruption during its placement and can be used in combination with other forms of internal fixation [1].

Linear external fixators (LEFs) are biomechanically versatile, allow simple assembly and disassembly, and are widely used to treat fractures [2].

Circular external fixators (CEFs) have some biomechanical advantages over linear ones and are used to treat fractures, limb length discrepancies, bone defects, non-unions or angular deformities. However, they need a high level of postoperative (PO) care and are sometimes uncomfortable for the patient because of the interference with muscle and joint motion, being usually cumbersome and heavy [3,4].

Hybrid linear-circular external fixators (HEFs) consist of linear skeletal fixation articulated with circular fixation components. These frames have been applied in growth deformities correction and also in fracture repair [5,6]; they are versatile and enable different configurations depending on the features of the bone and fracture.

HEFs share some of the positive characteristics of CEFs. The tensioned small-diameter wires allow the fixation of small bone fragments and counteract the bending or torsional displacements that are adverse for bone healing. Moreover, they enable the axial micro-motion, which stimulates callus formation and accelerates bone healing [7-9]. HEFs can also be changed in configuration, allowing PO adjustments and residual angular deformity correction [3,5]. CEFs usually require three or four rings to stabilize the fracture, while HEFs require a reduced number of rings due to their combination with the linear elements. As a consequence, HEFs are usually less cumbersome and are better tolerated by the patient, easier and less time consuming to apply [6,10]. This type of fixation is very useful in fractures with short juxta-articular fragments, in which AO/ASIF principles of a minimum of six cortices in each fragment [11] are difficult to achieve. The circular component contributes to a good stabilisation of the small fragment, while the linear element is used in the long one [5,10,12]. Ring diameter and thickness can be changed, while strut constructs can be added to the frame to adjust the axial stiffness of the fixator [13]. HEFs are currently used in several types of fractures, not only in juxta-articular but also transverse, oblique or comminuted diaphyseal fractures [10,12,14].

Published reports describe the use of HEFs for fracture stabilisation in dogs and cats [5,6,10,12] and a classification of these fixators has already been proposed [6,14]. The frame structures evaluated in this study did not fit completely with the existing classification, and for this reason it was extended to include all the potential combination of circular and linear elements.

The objectives of this study were to evaluate the clinical and radiographic outcome of fracture stabilization with HEFs in a cohort of clinical cases, and to propose an extended classification method for hybrid frames.

## Methods

Medical records of dogs and cats operated on for fracture stabilisation by HEFs at the institutions of the authors were reviewed. The use of external fixation was decided by the surgeon in charge of the case, and based on her/his experience and preference. The inclusion criteria were the availability of preoperative radiographs and PO rechecks until the time of frame removal. The HEF technique was defined as that involving an external fixator, whose components included at least one circular element in combination with at least a linear one. Sixty-eight fractures in 66 patients fulfilled the criteria for inclusion.

Animal signalment (age, breed, gender, and weight) and information about the bone involved and fracture location were recorded. The great variation in frame configurations observed during the inclusion phase prompted for an extended classification of the hybrid fixators.

Fractures were classified for location, if they were open or closed, and as simple (two bone fragments) or complex (more than two bone fragments).

The circular elements were 6-mm-thick aluminum alloy or carbon fiber rings 55, 85 and 115 mm in diameter, and were either complete (360°) or partial rings (270° or 180°) (Universal system, Ad Maiora, Cavriago, Italy). The rings were 15-mm wide and included 8-mm wide slots to place stabilizing elements, i.e. connection threaded bars, bolts for fixation of K-wires and clamps holding threaded pins. Linear uniplanar elements (rails) were made of 6-mm-thick aluminum alloy or thermoplastic material and were connected to the rings by nuts and washers, which could be flat or hemispheric. When flat nuts and washers were used, the linear element was locked in an orthogonal position with respect to the ring. When the hemispheric ones were employed, it could be inclined up to 30° with regards to the plane of the ring. Rails can have one, two or three oval slots with the same function as those of the rings. Linear cylindrical elements included rods and pin-holding cylinders. Rods were 80 or 110 mm in length, are made of aluminum alloy and can be placed perpendicular or oblique with respect to the ring they are connected with. They bring specifically designed clamps to hold threaded pins up to 4 mm in diameter. The cylinders had a diameter of 15 mm, are made of aluminum alloy and have holes that can hold up to three threaded pins that are locked by an interferential screw (Universal system, Ad Maiora, Cavriago, Italy). The carbon fiber rings and thermoplastic rails are radiolucent, and they were used to increase the fracture evaluation when the amount of metallic frame could have hindered it.

Details regarding the surgical procedure and configuration of the HEFs were recorded and an extended classification system was used to describe the frames used in this case series (Table 1).

**Table 1:** **Hybrid fixators classification based on rings and linear elements and their position in the frame**

	**1. Type I configuration**	
**IA**	1 ring and 1 linear element.	
**IB**	1 ring and 2 linear elements.	

**IC**	1 ring and 3 or more linear elements.	


	**2. Type II configuration**	
**IIA**	2 rings and 1 linear element.	

**IIB**	2 rings and 2 linear elements.	






**IIC**	2 rings and 3 linear elements	





**IID**	2 rings and 4 linear elements.	





	**3. Type III configuration**	
***IIIA:*** *3 or more rings and 1 linear element.*		
***IIIB*** *: 3 or more rings and 2 linear elements.*		
***IIIC*** *: 3 or more rings and 3 linear elements.*		
***IIID*** *: 3 or more rings and 4 or more linear elements.*		

Classification based on ring number was as follows.

I. one ring included in the frame.
II. two rings included in the frame.
III. three or more rings included in the frame.

Classification based on the linear element number was as follows.

A: One linear element included in the frame.
B: Two linear elements included in the frame.
C: Three linear elements included in the frame.
D: Four or more linear elements included in the frame.

For example, a construct IIC is composed of two rings and three linear elements.

Some of the frame configurations included strut constructs, made by one or two malleable stainless steel bars connecting the linear and circular elements by hooks [13]. Dynamizable linear components were used in some frames. They are linear elements that are composed of two telescopic rods that can be adjusted so as to compress or distract the fracture when in the locked position. They can also be set in a dynamic position, allowing axial loading during weight bearing, i.e. dynamization of the frame.

Fracture reduction, radiographic results and functional and cosmetic results were evaluated following a scale already described [3].

The fracture reduction was evaluated in the immediate PO, taking into account the contact between fragments and limb alignment:

- Excellent: 90 to 100% contact between fragments and angular deformity less than 5°.
- Good: 50 to 90% contact between fracture fragments or 5° to 10° angular deformity.
- Fair: 10 to 50% contact between fracture fragments or 10° to 30° angular deformity.
- Poor: contact between fracture fragments less than 10% or angular deformity greater than 30° [3].

Total fixator time, i.e. the time the fixator was left on the patient, destabilization, number of PO rechecks, system adjustments and minor and major complications were recorded.

The complications were considered minor when they were managed without additional surgery or they delayed the course of the treatment without influencing its expected outcome. Complications were considered major when they required additional surgery, substantial frame modification under general anesthesia or affected the final expected outcome.

Outcome was evaluated by radiographic, functional and cosmetic results at the latest available recheck. Radiographic results were graded as follows:

- Excellent: Fracture healed and angular deformity less than 5°.
- Good: Fracture healed and 5° to 10° angular deformity.
- Fair: Fracture healed and 10 to 30° of angular deformity.
- Poor: Fracture not healed or fracture healed and angular deformity greater than 30° [3].

Functional and cosmetic results were graded compared to the contralateral limb. When this was not possible, the evaluation was performed comparing the affected limb with a normal dog limb [3,15] and graded as follows:

- Excellent: Functionally normal and similar appearance to that of the contralateral normal limb.
- Good: Slight lameness only after exercise or minor difference with the contralateral normal limb.
- Fair: Slight to moderate lameness but consistent weight bearing or noticeable difference from the contralateral normal limb.
- Poor: Not weight bearing lameness or marked, disfiguring alteration compared to the contralateral normal limb.

The ordinal data collected were statistically analyzed with the average ± standard deviation, using the statistical package SAS 9.2 (2008 SAS Institute Inc., Cary, NC, USA). The Student’s t-test was used to evaluate the null hypothesis between the following variables:

- Type of fracture (open or closed, simple or complex) and healing time of the fracture.
- Presence of complications (minor or major) and healing time of the fracture.
- Presence of residual angular deformities and healing time of the fracture.

The chi-square test was also used to evaluate the null hypothesis between the following variables:

- Functional and cosmetic results and type of fracture (open or closed, simple or complex).
- Functional and cosmetic results and the presence of complications (minor or major).
- Type of fracture (open or closed, simple or complex) and presence of residual angular deformities.

The level of significance was set at P < .05 for all the tests.

The same statistical evaluations performed on the whole fractures population were repeated for radius-ulna and tibia fractures groups, which had the higher number of cases, enough to be statistically evaluated.

No previous permission was requested to an Ethical Committee, because the technique evaluated is already described in the veterinary literature and clinically applied on a regular basis.

## Results

A total of 68 fractures in 58 dogs and eight cats were retrospectively evaluated. Two dogs had bilateral fractures. Twenty-eight dogs were male and 30 female, while 4 cats were male and 4 female. Many breeds were included in the study.

Mean age was 3.2 ± 3.3 years (median, 2 years; range, 0.1 to 14 years) and mean body weight was 14 ± 10 kg for dogs (median, 9 kg; range, 1.5 to 45 kg) and 4 kg for cats (range, 3.1 to 5 kg).

Forty-three (63.2%) of the fractures were caused by car accidents, 11 (16.2%) by a fall from a height, 7 (10.3%) by unknown trauma, 3 (4.4%) by a jump during exercise, 3 (4.4%) by gunshot and 1 (1.5%) by a bite.

The average delay between fracture and treatment in cases that were not previously treated was 6 ± 4.9 days (median, 4 days; range, 1 to 20 days), while in the eight cases where the fracture was already operated on, the average delay between fracture and treatment was 33 ± 31.2 days (median, 15 days; range, 3 to 85 days).

Out of the 68 fractures, 35 (51%) were radio-ulna, 23 (34%) tibia, 6 (9%) humerus, 2 (3%) femur and 2 (3%) scapula fractures. Forty-four percent were right and 56% left limbs. Twenty-seven fractures (40%) were transverse, 24 (35%) were comminuted or multi-fragmentary, 11 (16%) were short oblique and 6 (9%) were long oblique or spiroidal. Twenty-seven (40%) fractures showed short peri or juxta-articular fragments, which were smaller than the 25% of the total length of the bone, and one fracture was articular.

Ten fractures were open.

Construct I was the most used frame structure in this study. Construct IA was applied in 16 (24%) cases, IB in 28 (41%) and IC in 7 (10%). Construct IIA was used in 2 (3%), IIB in 9 (13%), IIC in 2 (3%) and IID in 2 (3) cases. Constructs IIIB and IIIC were used in one case each.

A cancellous bone graft was performed in two cases and the frame was connected to an intramedullary pin (tie-in construct) in three cases.

Radiolucent elements were used in six frames. A radiolucent ring was used in five of them, while a radiolucent ring, bar and linear elements were used in the remaining one. Only one type of ring, i.e. full or partial, was employed in 61 cases. Full rings were used in 47 cases, 270° rings were used in six, and 180° rings in eight cases. Combinations of 180° rings with 360° rings were used in six cases, and 270° with 360° in one case. Linear uniplanar rails with one slot were included in 27 frame constructs, rails with two slots in 26 and rails with three slots in 4 frames. One frame included rails with one and three slots. The length of the rails was due to the size of the patient and to the features of the fracture. Cylinders were used in 4 frames, while cylindrical rods were used in 6 frames.

Thirty-three frame constructs (48%) included linear components positioned orthogonally, 31 (46%) positioned obliquely and 4 (6%) contained both orthogonal and oblique linear elements. In 45 (66%) cases, no strut construct was included in the frame, while in 16 (24%) a strut construct with one bar and in 7 (10%) a strut construct with 2 bars was applied. A dynamizable bar was employed in two cases.

Open minimally invasive surgery was performed in 45 (66%) fractures, fluoroscopy-assisted closed reduction was accomplished intraoperatively in 20 (30%), and in 3 (4%) cases closed reduction without fluoroscopy was carried out. Intraoperative skeletal traction for fracture reduction was used in 4 cases [16,17].

The mean time of surgery was 126 ± 41 minutes (median, 122 minutes; range, 45 to 220 minutes). The fracture reduction achieved in the immediate PO was excellent in 36 (53%) cases, good in 24 (35%) and fair in 8 (12%).

The mean time to fixator removal (fixator time) was 77 ± 32 days (median, 67 days; range, 21 to 167), and the average number of PO radiographic rechecks was 5 (range, 2 to 9).

No significant differences in treatment duration between simple or complex fractures (P = 0.11) or between open or closed fractures (P = 0.31) were found.

Destabilization was carried out in 27 (40%) cases. It was performed when bone callus was radiographically visible but it was not strong enough for complete fixator removal. In 21 cases a linear component was removed, while in five cases a circular element and in one case both linear and circular elements were removed. Frame adjustments were required in 16 cases (23%). Some adjustments were performed because of contact between the fixator and the skin, to exert compression on the fracture, dynamize the fracture callus or correct angular deformities.

Thirty-nine fractures (57%) presented minor complications, including serous discharge from the pins and wire tracts (n = 25), which led to sero-purulent discharge in two of these cases, distal limb edema (n = 5), hemorrhage from the pins and wire tracts (n = 3), intraoperative fractures (n = 2), pressure sores caused by the frame on the skin (n = 2), loss of tension of the wires (n = 2), and loosening of some elements of the fixator (n = 2). Out of these 39 patients, five presented two minor complications.

Twelve fractures (18%) presented major complications including osteomyelitis (n = 4), angular deformity that required revision (n = 2), secondary fracture (n = 2), bone sequestration (n = 1), frame instability (n = 1), elbow subluxation (n = 1) and quadriceps contracture (n = 1). There were no significant differences in treatment duration between cases that presented minor (*P =* 0.13) or major complications (P = 0.39) and those without complications.

Radiographic results at the time of frame removal was graded as excellent in 39 (57%) cases, good in 25 (37%) and fair in 4 (6%).

Functional and cosmetic results were scored as excellent in 46 (67%) cases, good in 18 (27%), fair in 3 (4%) and poor in one. In one of the cases, the radiographic outcome was graded as good, but the functional result was considered poor because of a quadriceps contracture. No statistically significant differences in functional and cosmetic results were found between simple or complex fractures (P = 0.49) or between open or closed fractures (P = 0.36). Moreover, no significant differences in functional and cosmetic results were observed between cases with minor (P = 0.84) or major complications (P = 0.18) and those without complications.

Residual angular deformities were present in 14 cases. Valgus deformity was seen in nine cases; three of them had 5°, two 8° and the remaining had 6°, 9°, 15° and 24° of valgus. Varus of 4° was present in one case, and 5° and 30° procurvatum in two. One case had 10° procurvatum combined with 10° supinatus, and another one 7° procurvatum combined with 15° valgus. Radius-ulna was the most prone to show residual angular deformities (8 cases), though this segment was overrepresented in this study.

Complex fractures did not show a significantly higher number of angular deformities (P = 0.19) than simple fractures. The time to fixator removal was significantly higher (P < .01) in cases with angular deformities compared to cases without.

### Radius-ulna

**Figure 1:**
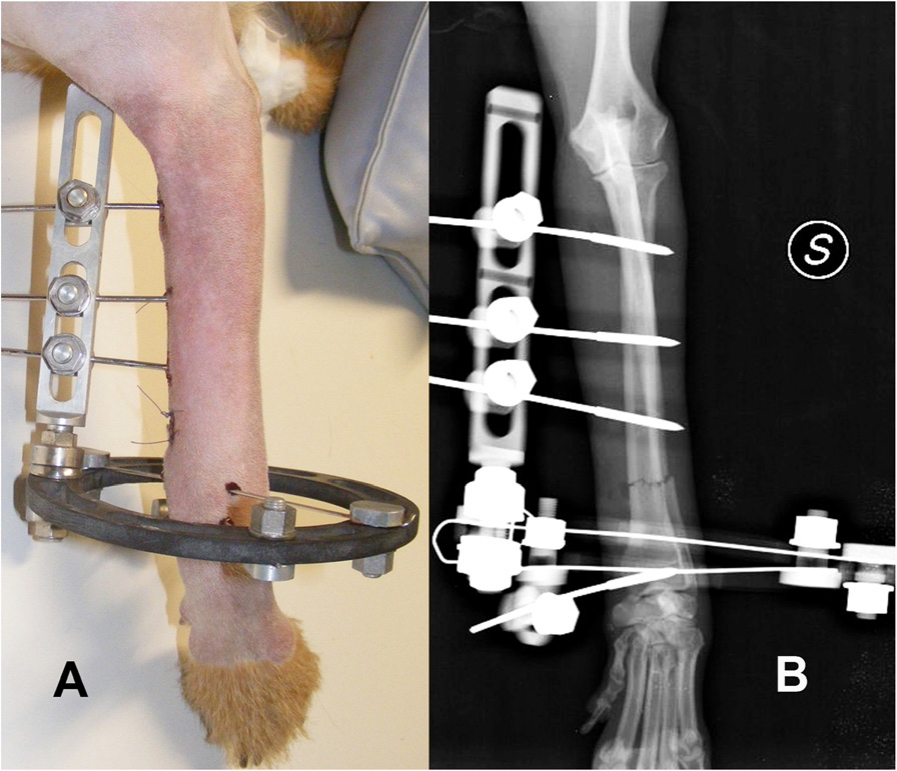
**Clinical (A) and radiographic (B) pictures of a construct IB for stabilisation of a radius-ulna fracture.** The distal short segment was stabilised by two K wires tensioned on the ring and a threaded pin on a post, and the proximal long segment by three threaded pins on a rail. Note the use of a radiolucent ring to allow for an unobstructed visualization of the fracture area.

Thirty-five cases were radius-ulna fractures: 22 (63%) were transverse, 7 (20%) comminuted, 4 (11%) oblique, and 2 (6%) spiroidal. The most used configurations were IB in 20 (57%) cases (Figure 1), IIB in 6 (17%) and IA in 3 (9%). Two of the remaining 6 cases (6%) were treated with configuration IIA, two with IC, one with IIIB and one with IID configuration. The rings used for radius-ulna fractures were 360° in 29 (83%) cases and 270° in 2 (6%); a combination of 360° rings distally and 270° and 180° rings proximally was used in 4 (11%) cases. Twenty-one (60%) frames contained orthogonal linear elements, 11 (31%) inclinable ones and 3 (9%) both of them. One or two-bar strut constructs were used in 8 (29%) constructs: one IA and 7 (35%) IB configurations. The healing time for radius-ulna fractures was 75.6 ± 30.6 days (median, 67 days; range, 36 to 167 days).

One case treated with construct IA, 10 (50%) cases treated with IB and 3 (50%) cases with IIB exhibited minor complications. None of the cases with IA had major complications, while 6 (30%) of IB and one of IIB showed major complications. No differences were observed in treatment duration between open and closed fractures (P = 0.69) or between simple and complex ones (P = 0.97). Time to fixator removal was significantly longer when minor complications were present (P < .01), while no difference in treatment duration was found when major complications were present (P = 0.17). There were no differences in the functional and cosmetic results between simple or complex fractures (P = 0.4) and between open or closed fractures (P = 0.5). No statistically significant differences in functional and cosmetic results were found when minor (P = 0.67) or major complications (P = 0.40) were present. Furthermore, there was no significant difference in functional results between cases that showed angular deformities and those that did not (P = 1), and also in the time to fixator removal when angular deformities were present (P = 0.17).

### Tibia

**Figure 2:**
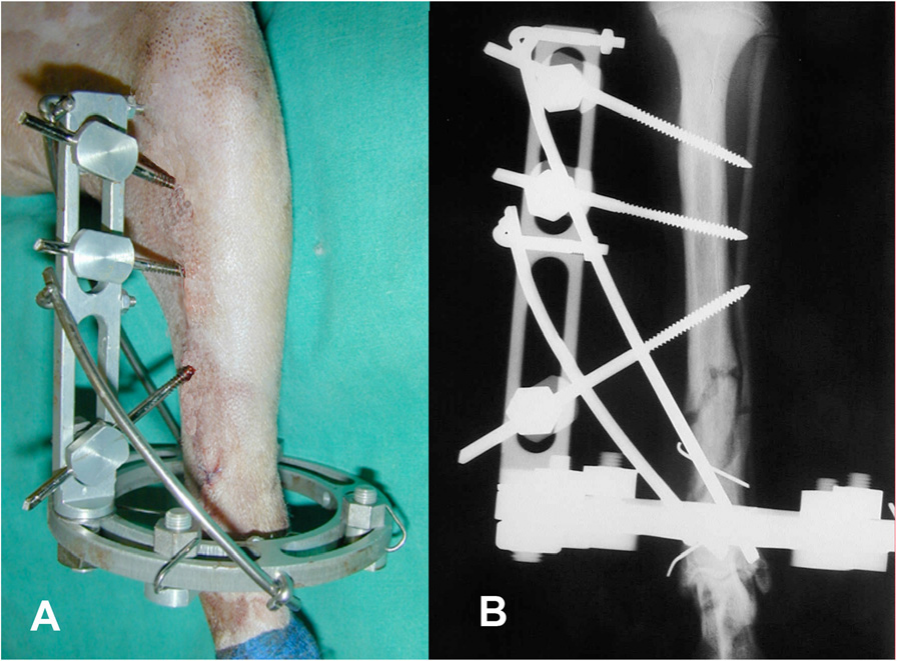
**Clinical (A) and radiographic (B) pictures of a construct IA for stabilisation of a tibia fracture.** The distal short segment was stabilised by two K wires tensioned on the ring and the proximal long segment was stabilized by three threaded pins on a rail. Note the use of two malleable steel bar connected to the linear and circular element of the frame as a strut construct.

Twenty-three cases included in the study were tibial fractures: 11 (48%) comminuted or multi-fragmentary, 3 (13%) transverse, 7 (30%) oblique and 2 (9%) spiroidal. The most used configurations were IA (Figure 2) in 10 (44%) cases, IB in 5 (22%) and IC in 4 (17%). In two of the remaining four cases construct IIC (9%), in one IIB and in one IID were applied.

The rings used were 360° in 17 (74%) cases, 270° in 3 (13 and 180° in one; a combination of 180° proximal ring with 360° distal ring was used in 2 (9%) cases. Inclinable linear elements were used in 14 (61%) cases, while 8 (35%) included orthogonal ones and one both of them. One or two-bar strut constructs were used in 12 (52%) cases. Time to fixator removal was 77.6 ± 39.2 days (median, 70 days; range, 21 to 164 days).

Seven (70%) the cases treated with the IA configuration had minor complications, whereas 3 (60%) cases treated by IB and 3 (75%) treated by IC configuration displayed minor complications. None of the cases with the IA configuration showed major complications; however, one case with IB and two cases with IC configuration had major complications.

No significant differences in treatment duration were found between simple or complex fractures (P = 0.11) and between open and closed ones (P = 0.4). Also, no significant differences were found in treatment duration when minor (P = 0.77) or major (P = 0.77) complications were present. There were no discernible differences in functional and cosmetic results between simple or complex fractures (P = 1) and between open and closed fractures (P = 0.6). Moreover, no differences in functional and cosmetic results were noted between cases with minor (P = 0.61) and major (P = 1) complications. There were also no differences in functional results between cases that showed angular deformities and those that did not (P = 0.6), and in the time to fixator removal when angular deformities were present (P = 0.14).

### Humerus

**Figure 3:**
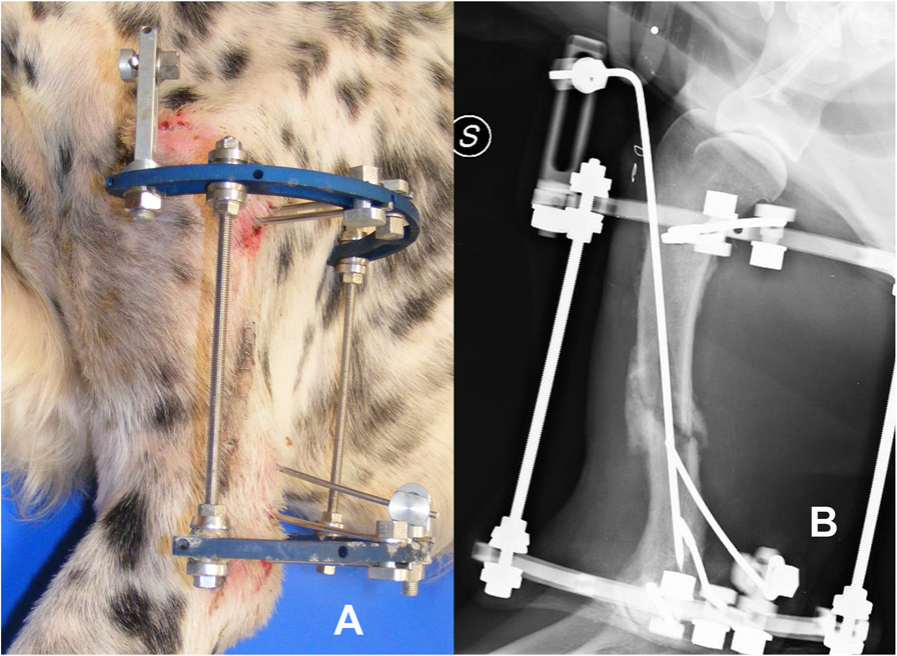
**Clinical (A) and radiographic (B) pictures of a construct IIB for stabilisation of a humeral fracture.** The distal fragment was stabilised by two threaded pins on the ring and a threaded pin on a post; the proximal segment was stabilised by two pins on the ring. An intramedullary pin is connected to a rail placed on the proximal ring, resulting in a tie-in configuration.

Six cases in the study were humeral fractures. Four were diaphyseal comminuted or multifragmentary fractures, one was spiroidal fracture and one was an articular, bicondilar fracture. Configuration IIB (Figure 3) was used in two cases, while configurations IA, IB, IC and IIIC were applied in one case each. The rings used in the frame construct were 180° in four cases, 270° in one and 360° in one. Four frames included inclinable linear elements and two orthogonal ones. No frame included a strut construct. Four cases had minor complications. None of the cases had major complications. Healing time for humeral fractures was 81.3 ± 23.6 days (median, 71.5 days; range, 60 to 120 days).

### Femur

Two cases involved femoral fractures; both of them were transversal fractures and treated with the IB configuration. Partial 180° rings were used in the IB configuration, one of the fractures included an inclinable linear element and the other an orthogonal one. No strut construct was employed in these frames. Healing time was 82.5 ± 31.8 days (median, 82.5 days; range, 60 to 105 days).

### Scapula

Two cases were scapular fractures; both of them were comminuted fractures and treated using the IA configuration with partial 180° rings. One of the frames included an inclinable linear element and the other an orthogonal one. A strut construct with two bars was employed in one of the cases. Healing time was 86.5 ± 33.2 days (median, 86.5 days; range, 63 to 110 days).

## Discussion

Hybrid external fixation is an emerging orthopaedic technique [1,5,10,12]. To date, a clear definition of what can be considered a hybrid fixator and a systematic classification of the frame structures used have not been proposed. While evaluating the cases for their inclusion in this study, we were prompted to define them in a simple yet consistent way and classify them in a clear, reproducible way, in order to create a homogenous group for evaluation. The classification used in this study is an extension of the existing one, and could be applied to the frame constructs that did not fit with the previous one.

Previous studies have described the use of HEFs mainly to treat radio-ulna and tibial fractures [5,12], while other study describe their application in femoral or humeral fractures [10]. The cases included in this study demonstrated their application to scapula fractures as well. This fixation technique is often recommended to manage fractures with short juxta-articular segments [10,12]. In this case series, a wide variety of fractures was treated, from simple transverse diaphyseal fractures to comminuted and peri-articular ones. Interestingly, the healing time did not significantly differ between simple and complex fractures, and between the quality of functional and cosmetic outcome.

Construct I was the most used frame structure in radius-ulna and tibial fractures. It was the easiest to apply, providing good stability to the fracture and being well tolerated by the patient. Construct IB was the most used in radius-ulna and IA in tibial fractures. The most used rings in tibial and radius-ulna configurations were 360° and 270°; when more than one ring was included in the frame, the half ring was always placed proximally, allowing flexion-extension of the stifle or elbow joint.

Few studies have reported the use of HEF for proximal limb fractures [5,10,12]. Construct IB was used in femoral fractures, using 180° rings and pins and wires in areas with less prominent muscle masses [6], despite a real safe corridor for pin insertion does not exist in the femur. Humeral fractures were treated with constructs IA, IB, IC and IIIC. In most cases, 180° and 270° rings were used and positioned laterally, in order to avoid interference with a correct elbow function; a 360° ring was used in a single case and elbow flexion was partially restricted.

Scapula fractures were treated with construct IA, using a 180° ring placed laterally, and was extremely well tolerated by the patients.

Inclinable linear elements using hemispheric nuts and washers facilitated fracture alignment in different planes and PO changes when necessary [6,18-20]. It has been shown that the use of hemispheric nuts and washers does not weaken the frame structure [19].

HEF can be combined with other fracture treatment techniques. In this case series, the most used technique used in combination with the fixator was intramedullary pinning in the tie-in configuration [5,10].

The use of radiolucent elements allowed for better assessment of fracture reduction and healing on radiographs [21,22]. Historically, PO radiographic rechecks were difficult with external fixators, especially with CEFs, because of the superposition of the fixator elements onto the fracture. Repeated exposures were required, often with some degree of obliquity to avoid interference from the fixator, with a subsequent increase in X-ray exposure and the time required by the procedure. Radiolucent elements dramatically reduce radiographic interference, as well as the time required for the recheck and X-ray exposure.

Once the primary bone callus has developed, axial micro-motion of the stabilized fracture segments stimulates further callus formation and bone healing [7-9]. For this purpose, both dynamization and destabilization techniques were used. Dynamizable fixators can be used as linear elements of the frame and can be changed to enable axial loading of the callus without torsional and bending forces because the fixator frame is not changed, other than for the sliding of the telescopic cylinders along the longitudinal axis. Destabilization can be achieved by removing some of the frame elements. Conceptually, this differs from dynamization because it introduces axial, torsional and bending forces in the callus, and not just the axial forces like with dynamization. Linear elements are removed for destabilization more frequently than circular ones in construct I frames because they stabilize the short fragment as the only holding element, while linear elements can be more than one, and they can be progressively removed. Furthermore, circular elements allow some micro-movements on the fracture callus, like compression and distraction, which are potentially beneficial for fracture consolidation and counteract more efficiently the bending and rotational forces that negatively affect callus maturation [7,8,23], and for this reason are usually the last elements to be removed. In this study, linear elements were removed in 78% of the cases where destabilization was performed. This may be due to the fact that 75% of the frames had just one ring, and it is much more likely to remove the lineal component before the ring.

Strut constructs were used to increase the axial stiffness of the frame. The addition of one or two malleable steel bars connected to the frame by custom designed hooks prevented the junction between the circular and linear elements from being a stress riser in the construct. This occurred by distributing the forces acting on this junction among various points instead of a single one, thereby decreasing the risk of breakage. A report described the use of two bars as a strut construct, which compared favorably with the single-bar construct [13]. In this study, 27% of the radius-ulna cases and 52% of the tibial cases employed strut constructs in their configuration. Moreover, this configuration promotes destabilization by removing the strut construct bars, thereby allowing more forces to be exerted on the fracture area.

Intraoperatively, fluoroscopy and minimally invasive surgical approaches were carried out. These techniques result in less devascularization and soft tissue damage. Intraoperative skeletal traction was used to reduce the fracture in some cases. The application of the methods for fracture stabilization can be greatly simplified when the fracture is held stably and reduced [15].

Frame preassembly was performed in most cases. It was based on preoperative radiographs and decreased surgical time and potential frame drawbacks [3]. As described in previous reports and observed in this case cohort, patient positioning was very important during surgery because it can affect residual angular deformities [3]. This can happen because in most instances the surgeon does not have a correct perspective view of the fractured limb. Based on the authors’ experience, the limb should be free to be moved along the all range of motion during surgery, to check for improper limb alignment and potential interference with the fixator frame. A high percentage of the patients showed minor complications related to inflammation and wire/pin tract serous or purulent discharge, as already described in previous reports [3,4,10]. In our study, 57% of the cases presented minor complications. These complications were controlled by thorough cleaning of the pin/wire bone interface, by antibiotics when osteomyelitis or soft tissue infection was suspected, and by removing the involved wire or pin in severe cases. In two cases, iatrogenic fractures were caused intraoperatively during pin insertion. One of them was caused by the pin placed too close to the fracture line, and the second one by the pin that opened a bone fissure that was not correctly appreciated by the surgeon. The offending pin was removed and the fracture stabilized by extending the frame structure, which stabilized both the original and iatrogenic fractures. This complication increased the healing time in radius-ulna fractures where it occurred.

Major complications occurred in 12 (18%) cases. Osteomyelitis was mainly due to preoperative contamination and soft-tissue trauma and was treated with local curettage, antibiotics and a cleaning protocol. In case of open fractures, HEFs can be useful because they do not hinder the wound, giving the opportunity for PO management. Other complications were represented by bone sequestration due to an avascular area of bone, bone lysis along wire and pin tracts, leading to frame instability, or nonunions. They were managed by revising the fracture area, removing fibrous and necrotic tissues, and increasing the stabilisation by changing the ineffective stabilizing elements.

Residual deformities were acutely or progressively corrected, thanks to the frame adjustments performed with the hemispheric nuts and washers [18-20]. Most of these changes were made with the awake patient, reducing the cost of the procedure and the risks associated with anesthesia [3]. In five (36%) of the 14 cases that had residual angular deformities this resulted in a longer healing time. In some cases the correction was not performed due to the unavailability of the owners, for example when they lived very far from the clinic, economical constraints, technical difficulties, or lack of appreciation of the problem by either the owners or the surgeon.

As already described for circular external fixation [3], the potential for PO adjustments of the frame and limb residual angular deformity corrections favorably altered the grade of the fracture in the immediate PO and at implant removal. Some of the cases that were classified as fair or good in the immediate PO were eventually classified as good or excellent following this PO correction.

The healing time in our case series was similar to those reported earlier [5,10], but longer in comparison with CEFs in antebrachial and crural fractures [3,4].

## Conclusions

Hybrid fixation represents a technique that can be considered for fracture stabilization, mostly for peri and juxta-articular fractures. It is a very versatile technique, and apparently the fracture features and complications do not heavily influence its outcome. For this reason they may be a suitable option for treatment of more demanding fractures. The extended classification system enables the inclusion of many potential frame configurations. Future biomechanical studies could be interesting to evaluate the influence of the relative position of linear and circular elements in the frame.

This study has some limitations. Being a retrospective clinical investigation, the study design has inherent uncontrolled variables. Among those is the multi-centric nature of the study, which introduces a high level of intra-surgeon variability in the surgical approach, PO management, complication management, and timing for dynamization and/or destabilization. Furthermore, the same surgeon who had operated on the fracture evaluated the outcome of the case, and this could have introduced bias on its objective evaluation.
